# Malignant Granular Cell Tumor of the Back: A Case Report and Review of the Literature

**DOI:** 10.1155/2014/794648

**Published:** 2014-11-24

**Authors:** Laura Stone McGuire, Danny Yakoub, Mecker G. Möller, Andrew Rosenberg, Alan Livingstone

**Affiliations:** ^1^Division of Surgical Oncology, University of Miami Miller School of Medicine, Jackson Memorial Hospital/Sylvester Comprehensive Cancer Center, 1120 NW 14th Street, CRB C232, Miami, FL 33136, USA; ^2^Sylvester Comprehensive Cancer Center, University of Miami Miller School of Medicine, Miami, FL 33136, USA; ^3^Department of Pathology, University of Miami Miller School of Medicine, Miami, FL 33136, USA

## Abstract

Malignant granular cell tumors are rare, intensely aggressive entities. This paper presents a case of a large rapidly recurrent malignant granular cell tumor with regional and distal metastases on the back of a 54-year-old Cuban man. The primary tumor recurred within six months of the original wide local excision and with satellite lesions apparent at twelve months, and the mass was diagnosed using the histological criteria established by Fanburg-Smith et al. for malignant granular cell tumors. By fifteen months, right axillary lymphadenopathy, multiple satellite lesions, pulmonary nodules, and distant metastasis in the right thigh were present. At sixteen months, wide local excision of recurrent mass and local satellite masses along with right axillary dissection and placement of Integra with subsequent split-thickness skin graft were performed by surgical oncology and plastic surgery teams. The surgical specimen measured 32.0 × 13.5 × 5.5 cm, containing multiple homogeneous masses with the largest mass 22.0 × 9.0 × 4.6 cm. Following surgery, patient was started on Pazopanib 800 mg/day based on phase III randomized trial data in the treatment of soft tissue sarcomas showing this as a potential novel therapy for malignant granular cell tumors.

## 1. Introduction

Granular cell tumors, first described by Abrikossoff in 1926 as myoblastomas [1], are tumors of Schwannian cell origin that may be classified as either benign or malignant. By convention, granular cell tumors are considered malignant when a morphologically benign granular cell tumor metastasizes to regional lymph nodes or to distant sites or causes death. Fanburg-Smith et al. further characterized malignant granular cell tumors histologically from their benign counterparts when their constituent cells met three out of six histopathologic criteria: necrosis, spindling, vesicular nuclei with large nucleoli, increased mitotic activity, high nuclear to cytoplasmic ratio, and pleomorphism [2, 3].

Granular cell tumors represent 0.5% of all soft tissue tumors [4], and the Malignant granular cell tumors (MGCT) are exceedingly rare representing less than 1-2% of all granular cell tumors [5, 6]. While both benign and malignant granular cell tumors present in a similar age range of 30–50 years, MGCT are more likely to affect African-Americans than whites and are two times more likely to occur in females than males [5]. In contrast to the smaller and slower-growing benign granular cell tumor, the malignant variant is fast-growing, has been reported to reach up to 15 cm in size, and has both a high rate of metastasis as well as short survival rates [5]. MGCT also typically develop in the lower extremity, often the thigh, whereas the benign tumors more commonly occur in the head and neck, most commonly the tongue [5]; however, granular cell tumors, both benign and malignant, have been found in a wide variety of locations, including skin [7], heart [8], lung [9], abdominal wall [10–12], pelvis [13], bladder [14], vulva [15], and esophagus [16].

While there are multiple case reports presently in the literature, representing a variety of presentations, this case involves a large rapidly recurrent malignant granular cell tumor of the skin and subcutaneous tissue of the back with regional and distant metastases, which demonstrates the capacity of these tumors to recur and to metastasize within a very limited amount of time. This case further details the surgical management for local disease control in this case and potential novel therapy for MGCT.

## 2. Case Report

**Figure 1:**
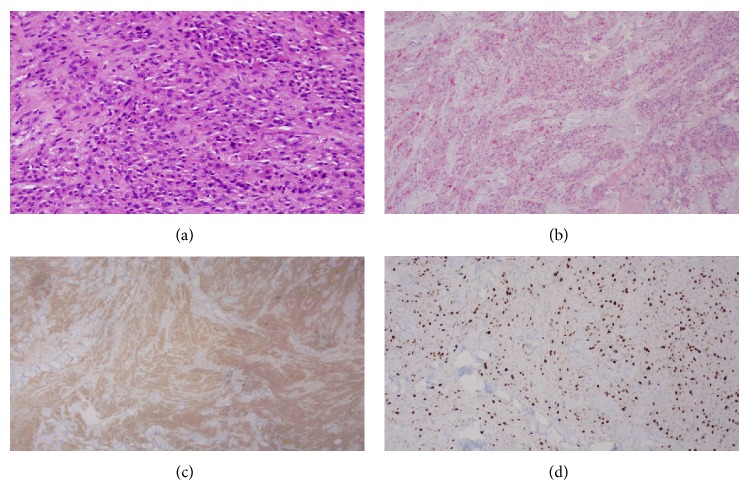
(a) High-power magnification of biopsy specimen (5 mitoses per 10 high power field), (b) S-100 immunohistochemistry stain of biopsy specimen, (c) CD68 immunohistochemistry stain of biopsy specimen, and (d) KI-67 proliferation marker of biopsy specimen.

**Figure 2:**
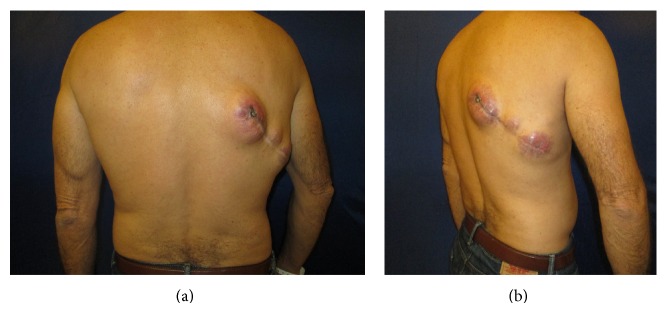
Preoperative assessment of recurrent malignant granular cell tumor at site of previous surgical excision.

This patient is a 54-year-old Cuban man with past medical history of emphysema, 105 pack-year smoking history, and family medical history of lung cancer who presented to Jackson Memorial Hospital with recurrent MGCT. The primary lesion first appeared on his right upper back in October 2011 and was excised in Cuba, and by April of 2012, the mass recurred at the original site with multiple satellite lesions in right axilla as well as right axillary lymphadenopathy (Figure 2). The Pathology Department (Dr. A.R.) reviewed the biopsy performed on the recurrent primary mass located on the right upper back in October 2012 at a local hospital and reported a diagnosis of malignant granular cell tumor, grade 2/3. The tumor was composed of aggregates and sheets of intermediate size spindle and polyhedral cells that had granular eosinophilic cytoplasm. The nuclei were vesicular and contained nucleoli; mitoses were numerous with 5 mitoses per high-power field; and scattered foci of necrosis were present (Figure 1). Immunohistochemistry was positive for S100 and CD68 and negative for keratin, EMA, CD34, MART1, and synaptophysin (Figure 1). The proliferation rate assessed by the KI-67 stain was 25% (Figure 1). CT of the chest with contrast performed on January 16, 2013, not only confirmed the recurrence of the primary tumor, involving surrounding skin and muscle, with an additional large mass in the right axilla and axillary lymphadenopathy, but also demonstrated multiple pulmonary nodules concerning for metastatic foci. CT of the abdomen and pelvis with contrast showed ill-defined hypodense intramuscular lesion in the anterior compartment of the right upper thigh, and a subsequent CT-guided biopsy of the lesion confirmed it to be metastatic malignant granular cell tumor.

**Figure 3:**
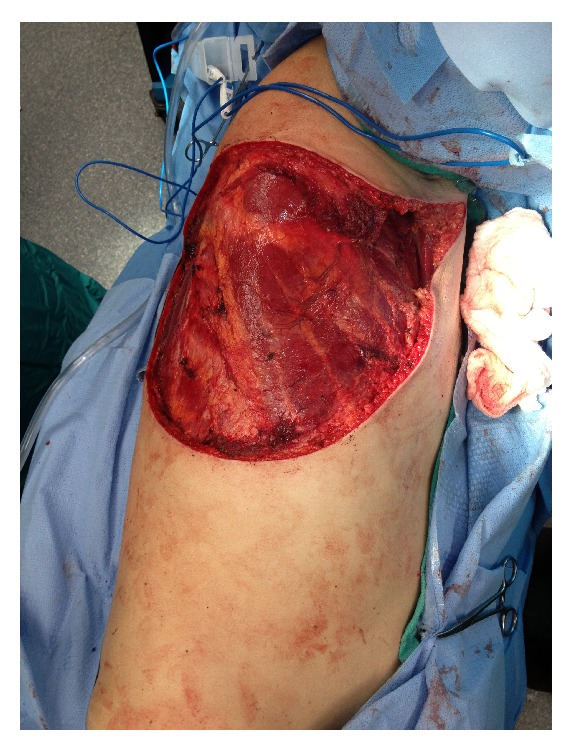
Intraoperative picture after wide local excision and right axillary lymph node dissection.

In February 2013, the patient was admitted back to the hospital as the mass on his back was extremely painful and was oozing serosanguinous fluid. Subsequently, he underwent a palliative resection of the recurrent primary tumor and satellite lesions on the upper right back as well as of the large matted right axillary nodal mass (Figure 3). Plastic surgery team used Integra dressings to cover the skin defects so as to ensure deep margin was free of tumor involvement upon final surgical pathology reporting.

Final surgical pathology indicated that the specimen on the right upper back was 32.0 × 13.5 × 5.5 cm, containing multiple homogeneous masses ranging from 2.0 × 2.0 × 2.0 cm to 22.0 × 9.0 × 4.6 cm. The large right axillary mass measured 10.0 × 8.0 × 4.3 cm. One out of five enlarged lymph nodes were positive for tumor. The deep margin was free of tumor.

**Figure 4:**
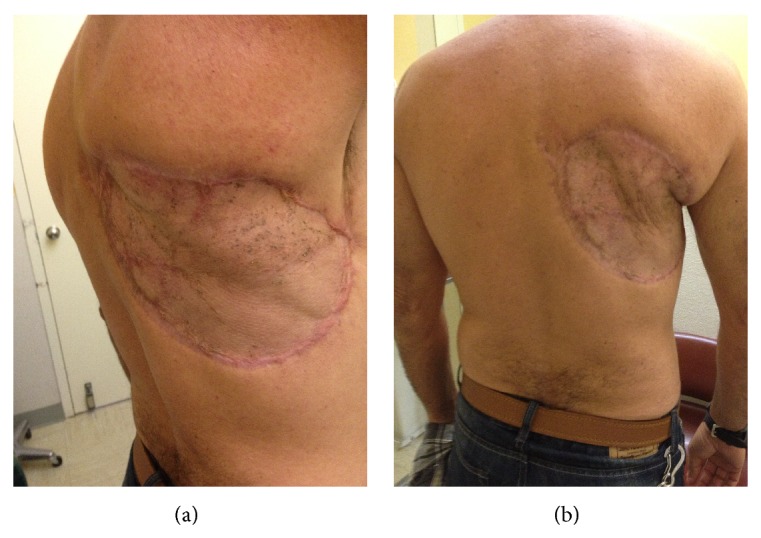
Postoperative visit status postsplit-thickness skin graft.

The postoperative course was uncomplicated, with development of clean granulation tissue under the Integra. The patient was brought back 3 weeks later when he underwent a split-thickness skin graft from the left upper thigh to the right upper back by the plastic surgery team. The graft take was 100% and the final result upon postoperative follow-up was satisfactory (Figure 4). A month after the reconstructive surgery, the patient was started on Pazopanib 800 mg/day monotherapy based on data from phase III studies in the treatment of soft tissue sarcomas suggesting improved survival [17]. The patient was seen six months after surgery with no evidence of local recurrence where he was decided to remain under surveillance as he continues his chemotherapy.

## 3. Discussion

Local recurrence and metastasis are relatively common in malignant granular cell tumors, with 32% rate of recurrence and 50% metastasis in the Fanburg-Smith analysis. Poor prognostic factors associated with MGCT include large tumor size, older patient age, increased mitotic activity, and Ki-67 greater than 10% [2, 3]. MGCT are fast-growing and can reach sizes of up to 15 cm [18]. Furthermore, cases with aggressive recurrence and rapid metastasis have been described in the literature [14, 15]. Distant and lymph node metastases are common, presenting between 3 and 37 months after initial diagnosis, and distal metastases often occur in the lung, liver, and bone [2, 3, 5, 19, 20]. Thus, consideration should be given to perform a sentinel lymph node biopsy at the time of the initial surgical resection. The differential diagnosis of MGCT includes a variety of different types of tumors, such as renal cell carcinoma, rhabdomyosarcoma, and alveolar soft part sarcoma; however, the diagnosis usually can be confirmed by assessment of the histomorphology and immunohistochemical profile.

In this case, the patient's primary tumor recurred within six months following first the tumor excision in Cuba. By one year, not only had the tumor recurred, but multiple satellite lesions were also evident. At fifteen months, imaging confirmed the recurrence and right axillary mass along with lymphadenopathy and asymptomatic metastases to the right upper thigh and lung. At the time of surgery at Jackson Memorial Hospital, sixteen months after the original resection, the recurrent tumor had grown to large proportions, with pathology noting the largest lesion, among several on the right upper back, was 22.0 × 9.0 × 4.6 cm, and the skin was ulcerating overlying the masses.

In addition to the incidence of recurrence and metastasis and the rapidity of tumor growth, other challenges exist in treating the malignant granular cell tumor. Currently, surgical resection remains the best possible option as no chemotherapy or radiation has been reported effective. Wide local resection is generally the treatment of choice; however, Mohs micrographic surgery has been utilized for malignant and benign granular cell tumors [21–26]. Although in this case, in addition to the wide local resection, Pazopanib was recently initiated as monotherapy. The patient continues to be monitored and so far there is no evidence of local recurrence and the response to this novel therapy continues to be monitored with promising initial results in this case. It is to be noted that the unavailability of medical records from this patient's care in Cuba limited our evaluation of the initial tumor and the features of the initial surgical treatment.
